# Volume de Gordura Epicárdica está Associada com Disfunção Endotelial, mas Não com Calcificação Coronariana: Do ELSA-Brasil

**DOI:** 10.36660/abc.20210750

**Published:** 2022-09-30

**Authors:** Karina P. M. P. Martins, Sandhi M. Barreto, Daniel Bos, Jesiana Pedrosa, Douglas R. M. Azevedo, Larissa Fortunato Araújo, Murilo Foppa, Bruce B. Duncan, Antonio Luiz P. Ribeiro, Luisa C. C. Brant

**Affiliations:** 1 Hospital das Clínicas Universidade Federal de Minas Gerais Belo Horizonte MG Brasil Hospital das Clínicas , Universidade Federal de Minas Gerais , Belo Horizonte , MG – Brasil; 2 Faculdade de Medicina Faculdade de Medicina Programa de Pós-Graduação Belo Horizonte MG Brasil Faculdade de Medicina , Programa de Pós-Graduação , Belo Horizonte , MG – Brasil; 3 Departamento de Medicina Social e Preventiva Universidade Federal de Minas Gerais Belo Horizonte MG Brasil Departamento de Medicina Social e Preventiva da Universidade Federal de Minas Gerais , Belo Horizonte , MG – Brasil; 4 Departamento de Epidemiologia Erasmus MC Holanda Departamento de Epidemiologia , Erasmus MC – Holanda; 5 Departamento de Radiologia e Medicina Nuclear Erasmus MC Holanda Departamento de Radiologia e Medicina Nuclear , Erasmus MC – Holanda; 6 Departamento de Epidemiologia Clínica Harvard TH Chan School of Public Health Boston EUA Departamento de Epidemiologia Clínica - Harvard TH Chan School of Public Health , Boston – EUA; 7 Departamento de Anatomia e Imagem Universidade Federal de Minas Gerais Belo Horizonte MG Brasil Departamento de Anatomia e Imagem da Universidade Federal de Minas Gerais , Belo Horizonte , MG – Brasil; 8 Departamento de Estatística Universidade Federal de Minas Gerais Belo Horizonte MG Brasil Departamento de Estatística , Interno, Universidade Federal de Minas Gerais , Belo Horizonte , MG – Brasil; 9 Secretaria de Saúde Comunitária Universidade Federal do Ceará Fortaleza CE Brasil Secretaria de Saúde Comunitária , Universidade Federal do Ceará , Fortaleza , CE – Brasil; 10 Hospital das Clínicas de Porto Alegre Universidade Federal do Rio Grande do Sul Porto Alegre RS Brasil Hospital das Clínicas de Porto Alegre, Universidade Federal do Rio Grande do Sul, Porto Alegre, RS – Brasil; 11 Programa de Pós-Graduação Universidade Federal do Rio Grande do Sul Porto Alegre RS Brasil Programa de Pós-Graduação, Universidade Federal do Rio Grande do Sul, Porto Alegre, RS – Brasil; 12 Departamento de Medicina Interna Universidade Federal de Minas Gerais Belo Horizonte MG Brasil Departamento de Medicina Interna, Universidade Federal de Minas Gerais, Belo Horizonte, MG – Brasil

**Keywords:** Aterosclerose, Gordura Intra-Abdominal, Obesidade Abdominal

## Abstract

**Fundamento:**

O aumento no volume de gordura epicárdica (VGE) está relacionado com doença arterial coronariana (DAC), independentemente de gordura visceral ou subcutânea. O mecanismo dessa associação não é claro. O escore de cálcio coronariano (CC) e a disfunção endotelial estão relacionados com eventos coronarianos, mas não está bem esclarecido se o VGE está relacionado com esses marcadores.

**Objetivos:**

Avaliar a associação entre VGE medido por método automatizado, fatores de risco cardiovasculares, escore de CC, e função endotelial. Métodos: Em 470 participantes do Estudo Longitudinal de Saúde do Adulto LSA-Brasil com medidas de VGE, escore de CC e função endotelial, realizamos modelos multivariados para avaliar a relação entre fatore de risco cardiovascular e VGE (variável resposta), e entre VGE (variável explicativa), e função endotelial ou escore de CC. Valor de p<0,05 bilateral foi considerado estatisticamente significativo.

**Resultados:**

A idade média foi 55 ± 8 anos, e 52,3% dos pacientes eram homens. O VGE médio foi 111mL (86-144), e a prevalência de escore de CC igual a zero foi 55%. Nas análises multivariadas, um VGE mais alto relacionou-se com sexo feminino, idade mais avançada, circunferência da cintura, e triglicerídeos (p<0,001 para todos). Um VGE mais alto foi associado com pior função endotelial: em comparação ao primeiro quartil, os valores de *odds ratio* para a amplitude de pulso basal foram (q2=1,22; IC95% 1,07-1,40; q3=1,50, IC95% 1,30-1,74; q4=1,50, IC95% 1,28-1,79) e para a razão de tonometria arterial periférica foram (q2=0,87; IC95% 0,81-0,95; q3=0,86, IC95% 0,79-0,94; q4=0,80, IC95% 0,73-0,89), mas não com escore de CC maior que zero.

**Conclusão:**

Um VGE mais alto associou-se com comprometimento da função endotelial, mas não com escore de CC. Os resultados sugerem que o VGE esteja relacionado ao desenvolvimento de DAC por uma via diferente da via do CC, possivelmente pela piora da disfunção endotelial e doença microvascular.

## Introdução

A gordura visceral é a deposição ectópica de gordura mais estudada, e a adiposidade visceral aumentada está relacionada à intolerância à glicose, resistência insulínica e doenças cardiovasculares, independentemente do índice de massa corporal (IMC). ^1^ A gordura epicárdica compartilha muitas das propriedades fisiopatológicas dos outros depósitos de gordura visceral, porém com efeitos potenciais adicionais sobre o processo aterosclerótico e inflamatório coronariano. ^2^ Pesquisadores do “ *The Framingham Heart Study* ^3 , 4^ e do “ *Multi-Ethnic Study of Atherosclerosis* ( *MESA* ) ^5 , 6^ estudaram a associação do volume de gordura epicárdica (VGE) com fatores de risco cardiovasculares, e identificaram que o VGE não só se correlaciona com obesidade e distúrbios metabólicos, como também com a presença de hipertensão e Doença Arterial Coronariana (DAC). Em uma revisão sistemática publicada em 2015, os autores descreveram nove estudos que avaliaram a capacidade do VGE em predizer eventos cardiovasculares maiores. Embora os achados não sejam consistentes para todos os estudos, a maioria sugere que a quantificação do VGE está significativamente associada com desfechos clínicos. ^7^

Estudos recentes mostraram que deposições de gordura epicárdicas estão associadas à DAC, mas não com escore de cálcio coronariano (CC), o qual avalia a calcificação nas artérias coronarianas e se mostrou associado com o risco de eventos cardiovasculares futuros em grandes estudos prospectivos. ^8^ Esses estudos sugeriram que o VGE poderia estar relacionado a outros mecanismos de formação de placas diferentes de placas calcificadas. ^9 , 10^ Nerlekar et al. ^11^ demonstraram, em uma metanálise publicada em 2017, a associação progressiva entre a presença de gordura epicárdica e placas ateroscleróticas de alto risco, ou seja, aquelas com elevado teor lipídico, pouca calcificação e uma fina capa fibrótica. ^11^ Outro estudo demonstrou que um VGE elevado foi associado à vulnerabilidade das placas nas artérias coronárias. ^12^

Nosso objetivo foi avaliar a associação entre VGE e fatores de risco cardiovasculares e marcadores subclínicos da aterosclerose – escore de CC e função endotelial microvascular, ambos preditores de eventos cardiovasculares. ^13 , 14^

## Métodos

### Participantes

**Figura 1:**
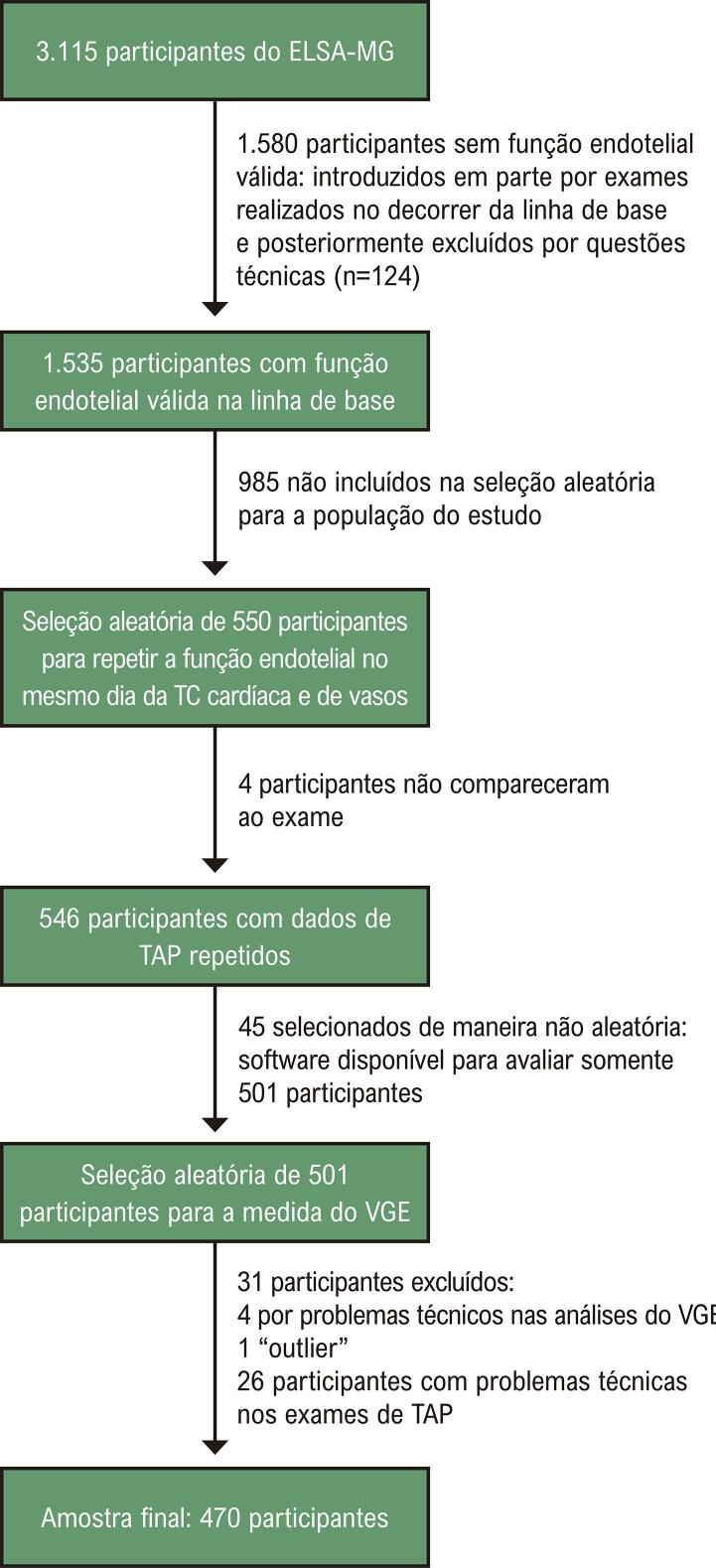
Fluxograma da seleção dos pacientes; TC: tomografia computadorizada; VGE: volume de gordura epicárdica; TAP: tonometria arterial periférica.

Nossa amostra incluiu participantes do Estudo Longitudinal de Saúde do Adulto (ELSA-Brasil), que tem como objetivo estudar determinantes de doença cardiovascular e diabetes em 15 105 adultos brasileiros. Os critérios de elegibilidade incluíram: funcionários ativos ou aposentados de cinco universidades e um instituto de pesquisa, com idade entre 35 e 74 anos, que se voluntariaram a participar do estudo. Outros detalhes do delineamento do estudo foram publicados previamente. ^15^ No centro de investigação do ELSA-Brasil em Minas Gerais (3115 participantes), foram realizadas tonometria arterial periférica (TAP) para avaliação da função endotelial, e tomografia computadorizada (TC) para avaliação do escore de CC. O exame de TAP foi introduzido no decorrer da linha de base, resultando em 1535 testes válidos. ^16^ Desses, 550 participantes foram aleatoriamente selecionados para reavaliação da TAP no mesmo dia da TC, e 546 realizaram o exame. Medidas do VGE foram realizadas em 501 participantes selecionados aleatoriamente com exames de TC e TAP válidos usando o programa R Development Core Team software (2020) R. Trinta pacientes foram excluídos devido a problemas técnicos nas análises do VGE (n=4) e da TAP (n=26), e um paciente com medida do VGE considerada *outlier* foi excluído, resultando em uma amostra final de 470 participantes ( Figura 1 ). ^16^ O ELSA-Brasil foi aprovado pelos comitês de ética das instituições participantes e pela Comissão Nacional de Ética em Pesquisa (CONEP 976/2006). Todos os participantes assinaram um termo de consentimento.

### Protocolo do estudo

Variáveis demográficas foram coletadas na linha de base do estudo e as características clínicas coletadas na segunda visita. Idade, sexo, raça autorrelatada, escolaridade, atividade física, obesidade, obesidade central, tabagismo, consumo de álcool, hipertensão, diabetes mellitus (DM), dislipidemia, hipertrigliceridemia, e escore de risco Framingham para DAC, ^17^ que estima a probabilidade de se desenvolver um evento coronariano em 10 anos, foram usados nas análises. A coleta de dados seguiu o protocolo do ELSA-Brasil, cujos detalhes podem ser encontrados em outras publicações. ^18 - 20^ Atividade física foi avaliada usando o questionário internacional de atividade física, na versão curta (IPAQ-SF, *International Physical Activity Questionnaire-short form* ). ^21^ No IPAQ-SF, cada tipo de atividade é ponderado por sua demanda de energia definida em MET (equivalente metabólico). O tempo gasto em atividade física por semana é então convertido em MET-minuto (MET-min/semana). O participante é considerado sedentário se a soma de MET-min/semana for menor 600; moderadamente ativo se a soma for 600-3000 MET-min/semana, e ativo se a soma for maior 3000MET-min/semana. Quanto a tabagismo, os participantes foram classificados como fumantes ou não fumantes e, em relação ao consumo de álcool, os participantes foram classificados em não usuários, com consumo moderado, ou com consumo excessivo (homens com consumo ≥ 210g álcool / semana e mulheres com consumo ≥ 140 g álcool / semana). Hipertensão foi determinada por relato do paciente, por pressão arterial sistólica (PAS) ≥ 140 mmHg, pressão arterial diastólica (PAD) ≥ 90 mmHg ou uso de medicamentos anti-hipertensivos. DM foi determinada por relato do paciente, uso de medicamento hipoglicemiante, glicemia de jejum ≥ 126 mg/dL, glicemia ≥ 200 mg/dL após duas horas de sobrecarga oral de glicose, ou hemoglobina glicada ≥ 6,5%. O escore de risco Framingham para DAC foi usado como uma variável categórica, e o risco cardiovascular estratificado em baixo (<10%), intermediário (10-20%) e alto (>20%).

### Avaliação do VGE

**Figura 2:**
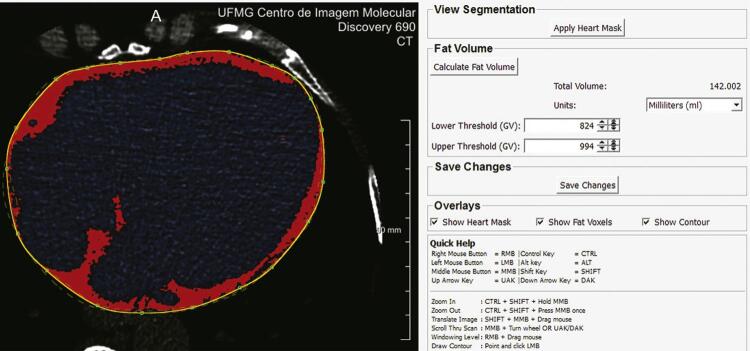
Calibração manual para avaliar o volume de gordura epicárdica em 15 participantes do ELSA-Brasil.

Foi realizada TC cardíaca sem contraste, sincronizada ao ECG, para avaliar CC e VGE, usando um tomógrafo de 64 canais (Lightspeed, General Electric). As imagens foram adquiridas durante apneia respiratória por 8-12 segundos. O VGE foi quantificado utilizando um método automatizado, validado, padronizado por Shahzad et al. ^22^ Em resumo, o método incluiu duas fases: (1) segmentação do coração e (2) quantificação do VGE em mL. A segmentação do coração foi realizada usando o multi-atlas, e registrada utilizando o programa Elastix, descrito por Klein et al. ^23^ Para a quantificação do VGE, adotou-se uma escala entre -30 e -200 unidades Hounsfield. Foi realizada uma calibração manual para o presente estudo, usando o programa MeVisLab para a delimitação manual do pericárdio de 15 participantes ( Figura 2 ). Os resultados foram comparados aos obtidos pelo programa Elastic, e calibrados.

### Medidas do Escore de CC

As imagens foram transferidas para a *workstation* (GE ADW 4,5) e ao servidor de imagens do ELSA-Brasil, em que o escore de CC foi calculado pelo método Agatston por um radiologista com 10 anos de experiência, cego quanto às informações clínicas dos pacientes.

### Medidas de função endotelial

O exame de TAP foi realizado por dois examinadores certificados, usando o aparelho Endo-PT2000 (Itamar Medical Ltd., Cesareia, Israel), no mesmo dia da TC. ^16 , 24^ Em resumo, o manguito foi colocado no braço não dominante do participante, 2 cm acima da fossa cubital, e as sondas posicionadas em cada dedo indicador. A amplitude de pulso basal (APB) foi medida por cinco minutos. O fluxo arterial foi interrompido em um lado por cinco minutos inflando o manguito à pressão suprassistólica. Após cinco minutos, realizou-se a deflação do manguito para induzir hiperemia reativa, e o sinal da TAP foi registrado por mais cinco minutos. O dedo contralateral foi usado para controle das alterações sistêmicas. Foram usadas duas variáveis da TAP: APB média, que reflete o tônus vascular basal e é calculado por transformação logarítmica dos valores médios de APB de ambos os braços, e a razão TAP, que reflete a resposta à hiperemia reativa. A razão TAP é a razão entre a amplitude de pulso 90-120 segundos após deflação do manguito e a APB média. O resultado é dividido pela razão correspondente obtida do dedo controle e transformado ao seu logaritmo natural.

### Análise estatística

As variáveis categóricas foram expressas em frequências e porcentagens, e as variáveis contínuas em média ± desvio padrão ou mediana e intervalo interquartil, de acordo com o resultado do teste de Kolmogorov-Smirnov. Devido ao desvio à esquerda da distribuição do VGE, o logaritmo natural do VGE foi usado nas análises em que o VGE foi a variável dependente (associação com fatores de risco cardiovasculares). Nas análises em que o VGE foi a variável independente, foram construídos quartis do VGE (associação com medidas subclínicas da aterosclerose: escore de CC e função endotelial). O escore de CC foi dicotomizado em 0 ou > 0, e as medidas de função endotelial foram analisadas como variáveis contínuas.

As análises estatísticas foram realizadas em três etapas e por modelos adaptados à distribuição das variáveis de resposta: 1- avalição da associação univariada e multivariada entre fatores de risco cardiovasculares e VGE por regressão linear; 2- avaliação da associação univariada e multivariada entre VGE e escore de CC por regressão logística; e 3- avaliação da associação univariada e multivariada entre VGE e medidas da função endotelial por regressão linear.

As variáveis foram consideradas em quatro modelos multivariados definidos *a priori* , e mantidas se mostrassem associação nas análises univariadas, com p<0,10, como a seguir: Modelo 1, ajustado para sexo e idade; Modelo 2: Modelo 1, mais raça e escolaridade; Modelo 3: Modelo 2, mais atividade física, IMC, circunferência da cintura, e tabagismo; Modelo 4: Modelo 3, mais PAS, uso de medicamentos anti-hipertensivos, DM, colesterol total/HDL, e triglicerídeos.

O IMC e a circunferência da cintura não foram incluídos simultaneamente nos modelos 3 e 4, devido à colinearidade com fator de inflação da variância (FIV) próximo a 8. Quando ambos eram estatisticamente significativos, a circunferência da cintura era incluída, por ser uma medida de gordura ectópica, como o VGE. O escore de risco Framingham para DAC foi analisado separadamente, uma vez que esse já representa uma avaliação do risco de DAC incorporando o efeito combinado de vários fatores de risco cardiovasculares.

Um valor de p<0,05 bilateral foi considerado estatisticamente significativo. Devido aos números de variáveis no modelo, aplicou-se a correção de Bonferroni e um valor de p<0,038 foi considerado estatisticamente significativo. Todas as análises foram realizadas utilizando o programa R Development Core Team (2020).

## Resultados

A Tabela 1 apresenta as características dos participantes. A idade média foi de 55 ± 8 anos, e 52,3% eram homens. Quanto à raça, 50,9% eram brancos, 33,0% pardos, e 12,3% negros. Observou-se uma alta proporção de participantes sedentários (59,2%) e de alto nível educacional, o qual refletiu o tipo de trabalho dos participantes (servidores de universidades). O IMC médio foi 26,9 ± 4,6 kg/m ^2^ e a circunferência da cintura mediana foi 92 (84-101) cm. O VGE mediano foi 111 (IQ 86-144) mL. Um escore de CC igual a zero foi detectado em 261 (55,5%) participantes. A APB média foi 6,57 ± 0,62, e a razão TAP média foi 0,42 ± 0,34.

**Tabela 1:** Características dos participantes do estudo (N = 470)

Características	
**Idade, anos**	55 ± 8
**Sexo masculino %**	246 (52,3)
Raça*, %	
Negra	58 (12,3)
Parda	239 (50,9)
Branca	155 (33)
**Nível educacional, %**	
Ensino fundamental incompleto	10 (2,1)
Ensino fundamental	17 (3,6)
Ensino fundamental completo	87 (18,5)
**Ensino médio completo**	356 (75,7)
**Ensino superior**	
**Status de atividade física, %**	
Sedentários	278 (59,1)
Moderadamente ativos	172 (36,6)
Ativos	20 (4,6)
**Fumantes,** %	34 (7,2)
**Consumo de álcool excessivo,** %	48 (10,2)
**IMC,** kg/m ^2^	26,9 ± 4,7
**Circunferência da cintura,** cm	91,8 (84,4 – 100,7)
**Diabetes mellitus,** %	81 (17,2)
**Hipertensão,** %	183 (38,9)
**PAS,** mmHg	121 ± 16
**Tratamento para hipertensão,** %	159 (33,8)
**Colesterol total/HDL**	3,84 ± 0,96
**Triglicerídeos,** mg/dL	108 (79 – 155)
**ECC = 0†,** %	261 (55,5)
**VGE,** mL	111 (86 -144)
**APB**	657 ± 0,62
**Razão TAP**	0,42 + 0,34

*Treze pacientes foram excluídos por representarem uma amostra pequena (raça amarela e indígena), cinco participantes não proveram os dados, e † um participante não possuía dados. IMC: índice de massa corporal; APB: amplitude de pulso basal; VGE: volume de gordura epicárdica; TAP: tonometria arterial periférica; PAS: pressão arterial sistólica; ECC: escore de cálcio coronariano; HDL: lipoproteína de alta densidade.

### Associação entre fatores de risco cardiovascular e VGE

A associação univariada entre fatores de risco cardiovascular e VGE está apresentada na Tabela Suplementar 1 . Como o VGE foi transformado em seu logaritmo natural, um aumento de 0,1 no coeficiente de cada variável explicativa indica um aumento de 10,5% no VGE. Somente tabagismo, atividade física, e escolaridade não mostraram associação significativa com VGE. Considerando raça/cor de pele, indivíduos da raça negra e pardos apresentaram VGE significativamente mais baixo que indivíduos da raça branca. Um aumento no VGE foi observado com a progressão de risco cardiovascular avaliado pelo escore de risco Framingham para DAC ( Figura Suplementar 1 ).

Na análise multivariada ( Tabela 2 ), as seguintes covariáveis mantiveram-se associadas com um VGE mais alto: sexo masculino, idade mais avançada, circunferência da cintura, e triglicerídeos. No modelo final, a raça negra manteve-se associada a um VGE mais baixo.

**Tabela 2:** Modelos de regressão linear da associação entre fatores de risco cardiovascular e volume de gordura epicárdica

Variável	Modelo 1		Modelo 2		Modelo 3		Modelo 4	

	β	IC95%	β	IC95%	β	IC95%	β	IC95%
Idade	1,01	(1,01 – 1,02) †	1,01	(1,01 – 1,02) †	1,01	(1,007 – 1,013) †	1,01	(1,01 – 1,02) †
**Sexo** (referência: homens)	0,76	(0,71 – 0,82) †	0,77	(0,72 – 0,82) †	0,87	(0,82 – 0,93) †	0,87	(0,81 – 0,93) †
**Raça** (referência: branca)								
Negra	,,,	,,,	0,85	(0,77 – 0,95)*	0,83	(0,76 – 0,91) †	0,85	(0,77 – 0,93) †
Parda	,,,	,,,	0,92	(0,86 -0,99)*	0,93	(0,88 – 1,00)*	0,94	(0,88 -1,00)
**Circunferência da cintura**	,,,	,,,	,,,	,,,	1,02	(1,01 – 1,02) †	1,02	(1,01 – 1,02) †
**Consumo de álcool excessivo**					1,06	(0,97 – 1,17)	1,05	(0,95 – 1,15)
**Diabetes Mellitus**	,,,	,,,	,,,	,,,	,,,	,,,	0,96	(0,88 – 1,04)
**PAS**	,,,	,,,	,,,	,,,	,,,	,,,	1,00	(0,996 – 1,001)
**Tratamento para hipertensão**	,,,	,,,	,,,	,,,	,,,	,,,	0,99	(0,92 – 1,06)
**Colesterol Total/HDL**	,,,	,,,	,,,	,,,	,,,	,,,	0,98	(0,95 – 1,02)
**Triglicerídeos**	,,,	,,,	,,,	,,,	,,,	,,,	1,00	(1,000 - 1,001)*

* p<0,05, †p< 0,001. β coeficiente de regressão exponencial, IC: intervalo de confiança; PAS: pressão arterial sistólica; HDL: lipoproteína de alta densidade.

### Associação entre VGE e escore de CC

Em relação à associação entre VGE e CC, a análise logística bruta revelou maiores chances de um escore de CC maior que zero entre indivíduos no terceiro e no quarto quartis do VGE. No entanto, essas associações perderam significância estatística na análise multivariada ( Tabela 3 ) em todos os modelos considerados. A análise univariada do escore de CC com fatores de risco cardiovascular está apresentada na Tabela Suplementar 2 .

**Tabela 3:** Modelos de regressão logística da associação entre quartis do volume de gordura epicárdica e presença de cálcio coronariano

Variável	OR	IC 95%	Valor p
**Modelo 1** (referência: primeiro quartil)			
Segundo quartil	1,07	(0,60 – 1,91)	0,823
Terceiro quartil	1,19	(0,66 – 2,14)	0,565
Quarto quartil	1,72	(0,93 – 3,19)	0,082
**Modelo 2** (referência: primeiro quartil)			
Segundo quartil	0,99	(0,54 – 1,80)	0,966
Terceiro quartil	1,03	(0,56 – 1,90)	0,921
Quarto quartil	1,48	(0,79 -2,79)	0,221
**Modelo 3** (referência: primeiro quartil)			
Segundo quartil	0,91	(0,50 – 1,68)	0,776
Terceiro quartil	0,78	(0,41 – 1,49)	0,455
Quarto quartil	0,87	(0,41 – 1 ,52)	0,709
**Modelo 4** (referência: primeiro quartil)			
Segundo quartil	0,94	(0,50 – 1,74)	0,838
Terceiro quartil	0,81	(0,42 – 1,59)	0,547
Quarto quartil	0,88	(0,41 – 1,87)	0,734

*OR Odds Ratio IC: intervalo de confiança. Primeiro quartil (22,8-86,2), segundo quartil (86,2-112), terceiro quartil (112-144), quarto quartil (144-331). Modelo 1: sexo, idade. Modelo 2: Modelo 1, mais raça e escolaridade. Modelo 3: Model 2, mais nível de atividade física, IMC, circunferência da cintura, e tabagismo. Modelo 4: Modelo 3, mais PAS, uso de anti-hipertensivos, DM, colesterol total / HDL e triglicerídeos.*

### Associação entre VGE e função endotelial

**Figura 3:**
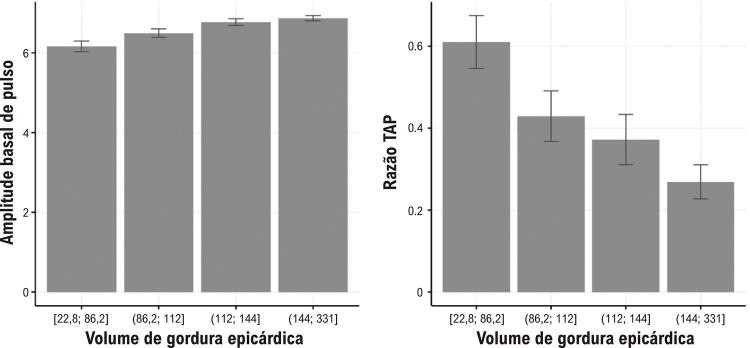
Medidas de função endotelial (médias) de acordo com o volume de gordura epicárdica estratificada em quartis; TAP: tonometria arterial periférica.

Na associação univariada ( Tabela Suplementar 3 ), observamos uma associação estatisticamente significativa de todos os quartis de VGE com as medidas de função endotelial. Também identificamos um gradiente de dose resposta para os quartis de VGE e as medidas de função endotelial: a APB média foi progressivamente mais alta e a razão TAP mais baixa – refletindo maior disfunção endotelial – nos quartis de VGE mais altos ( Figura 3 ). Na análise multivariada, a associação continuou estatisticamente significativa em todos os modelos ( Tabela 4 ).

**Tabela 4:** Modelos de regressão logística da associação entre volume de gordura epicárdica e função endotelial

Variável	Amplitude de pulso basal	Valor p	Razão PAT	Valor p
	OR (IC 95%)		OR (IC 95%)	
**Modelo 1 (referência: primeiro quartil)**				
Segundo quartil	1,31 (1,14 – 1,49)	0,001	0,86 (0,79 – 0,93)	<0,001
Terceiro quartil	1,63 (1,42 – 1,87)	< 0,001	0,83 (0,76 – 0,89)	<0,001
Quarto quartil	1,62 (1,40 – 1,88)	< 0,001	0,77 (0,71 – 0,84)	<0,001
**Modelo 2 (referência: primeiro quartil)**				
Segundo quartil	1,22 (1,07 – 1,40)	0,003	0,87 (0,81 – 0,94)	<0,001
Terceiro quartil	1,54 (1,35 – 1,77)	< 0,001	0,84 (0,77 – 0,91)	<0,001
Quarto quartil	1,54 (1,33 – 1,78)	< 0,001	0,79 (0,73 – 0,86)	<0,001
**Modelo 3 (referência: primeiro quartil)**				
Segundo quartil	1,22 (1,06 – 1,39)	0,004	0,88 (0,81 – 0,95)	0,001
Terceiro quartil	1,52 (1,32 – 1,75)	< 0,001	0,85 (0,78 – 0,92)	<0,001
Quarto quartil	1,49 (1,26 – 1,77)	< 0,001	0,80 (0,73 – 0,89)	<0,001
**Modelo 4 (referência: primeiro quartil)**				
Segundo quartil	1,22 (1,07 – 1,40)	0,004	0,87 (0,81 – 0,95)	0,001
Terceiro quartil	1,50 (1,30 – 1,74)	< 0,001	0,86 (0,79 – 0,94)	<0,001
Quarto quartil	1,50 (1,28 – 1,79)	< 0,001	0,80 (0,73 – 0,89)	<0,001

*OR OR Odds Ratio IC: intervalo de confiança. Primeiro quartil (22,8-86,2), segundo quartil (86,2-112), terceiro quartil (112-144), quarto quartil (144-331). Modelo 1: sexo, idade. Modelo 2: Modelo 1, mais raça e escolaridade. Modelo 3: Model 2, mais nível de atividade física, IMC, circunferência da cintura, e tabagismo. Modelo 4: Modelo 3, mais PAS, uso de anti-hipertensivos, DM, colesterol total / HDL e triglicerídeos.*

## Discussão

O presente estudo avaliou o VGE por meio de um método automatizado e sua associação com fatores de risco cardiovasculares e marcadores subclínicos da aterosclerose – escore de CC e função endotelial em 470 participantes do ELSA-Brasil. Os principais achados foram: 1) associação do VGE com a maioria dos fatores de risco na análise multivariada, um VGE mais alto foi encontrado para: sexo masculino, idade mais avançada, raça branca, e níveis mais altos de triglicerídeos e de circunferência da cintura; 2) VGE não foi associado com a presença de CC nos modelos multivariados; 3) VGE aumentado foi associado com disfunção endotelial nos modelos multivariados, de maneira dose-resposta. Nossos achados geram a hipótese de que deposições de gordura epicárdica podem estar associados à DAC por uma via distinta à de placas calcificadas, e potencialmente relacionada à disfunção endotelial, doença microvascular, e possível predominância de placas lipídicas não calcificadas.

Primeiramente, o valor mediano de VGE foi 111 (86-144) mL, foi comparável aos resultados observados por Bos et al. 101 (80-130), ^25^ e no *Framingham Heart Study* (108 ± 40) mL, ^10^ sugerindo que, embora a transição nutricional esteja em um estágio ligeiramente atrasado no Brasil, em comparação a países europeus e nos EUA, deposições de gordura ectópica parecem estar presentes de maneira similar. Bos et al., ^25^ utilizando o mesmo método automatizado descrito por Shahzad et al., ^22^ avaliaram a associação entre VGE, presença de calcificação nos leitos vasculares e fatores de risco cardiovasculares, em uma análise transversal. Os autores observaram que um aumento no VGE associou-se com um aumento no volume de calcificação na artéria coronária e na artéria carótida externa, mas somente em homens [diferença no volume de calcificação com aumento de um desvio padrão do VGE: 0,12 (IC95%: 0,04; 0,19) e 0,14 (IC95%: 0,06; 0,22), respectivamente]. ^25^ Não encontramos associação entre VGE e escore de CC após ajuste quanto aos fatores de risco. O perfil distinto das populações estudadas pode explicar as diferenças, uma vez que um maior número de mulheres, com idade mais avançada, foi avaliado por Bos et al. Quanto aos resultados, não realizamos uma análise estratificada por sexo, devido ao nosso menor tamanho amostral.

Em uma publicação mais recente de Lee et al. ^10^ do Framingham Heart Study, a associação entre VGE e CC foi avaliada longitudinalmente ^10^ em 1732 participantes do *Offspring and Third Generation Cohorts* (49,6% homens, idade média 49,9 anos), acompanhados por 6,1 anos. O estudo avaliou 1024 participantes com escore de CC basal igual a zero, e 708 participantes com escore de CC basal maior que zero. Não observamos associação entre o aumento no VGE e a progressão do escore de CC após ajuste quanto ao IMC, circunferência da cintura e tecido adiposo visceral, ou entre CC incidente e VGE após ajuste quanto as variáveis clínicas. ^10^ A ausência de associação relatada aqui também foi descrita em uma metanálise recente publicada por Mancio et al., ^9^ que demonstrou que a associação entre VGE e CC não foi mantida em modelos multivariados, mas um VGE mais alto permaneceu associado com estenose obstrutiva ou estenose coronária importante, e eventos cardiovasculares adversos maiores. ^9^ A hipótese dos autores é a de que o VGE esteja associado com DAC por outros mecanismos e formas de apresentação que se diferem do efeito das placas calcificadas. Outra hipótese possível é a de que o mecanismo de associação entre VGE e DAC possa expressar diferentes momentos na história natural da doença, sendo mais precoce em comparação à expressão de cálcio coronariano. ^26^

Para melhor entender o mecanismo pelo qual a gordura epicárdica e a DAC possa estar relacionado, nós investigamos a associação entre VGE e função endotelial microvascular. ^27^ Encontramos que um VGE mais alto foi fortemente associado com comprometimento da função microvascular, mesmo em modelos multivariados. A associação entre disfunção endotelial e VGE mais alto foi demonstrada em estudos prévios. Contudo, enquanto todos esses estudos tenham utilizado dilatação mediada pelo fluxo (DMF), ^28 - 32^ o método usado para avaliar função endotelial no ELSA-MG foi TAP. A DMF difere-se da TAP nos vasos em que a função endotelial é avaliada – enquanto a DMF a avalia na artéria braquial – um vaso condutor – a TAP a avaliar na microvasculatura. ^14 , 33 , 34^ Considerando que a disfunção endotelial é um preditor de eventos cardiovasculares, ^33 , 35^ nossos resultados apoiam a hipótese de que um VGE mais alto esteja relacionado com DAC por vias diferentes da formação de placas ateroscleróticas calcificadas, incluindo disfunção endotelial, doença microvascular, e placas lipídicas não calcificadas. Devido à proximidade da gordura epicárdica às artérias coronárias, os tecidos de gordura epicárdica podem exercer efeitos parácrinas sobre os vasos, em que mediadores inflamatórios produzidos pela gordura epicárdica atuam sobre os vasos, levando à disfunção endotelial. ^11^ Dada a possibilidade de nossos resultados representarem um epifenômeno, desenvolvemos modelos na tentativa de minimizar esse efeito ajustando-se quanto às variáveis de confusão.

Nosso estudo tem algumas limitações. Este é um estudo transversal que não permite inferências sobre causalidade. No entanto, este estudo foi incluído em um estudo coorte, e o acompanhamento dos participantes quanto a eventos cardiovasculares maiores seria possível em outras publicações. O tamanho da amostra não permitiu análise de subgrupos estratificados por sexo ou obesidade, uma vez que os indivíduos representam parte da amostra da grande coorte do estudo ELSA. Ainda, somente a função endotelial microvascular foi estudada, e sua avaliação em outros leitos arteriais poderia complementar nossos achados. No entanto, a função endotelial microvascular correlaciona-se mais fortemente com fatores de risco cardiovasculares metabólicos ^14 , 34^ – os quais, por sua vez, estão mais intimamente relacionados a fenótipos de obesidade – em comparação à função endotelial avaliada nos vasos condutores. ^34^ Além disso, nós não utilizamos o método padrão ouro para avaliar função endotelial, por esse ser um método invasivo. Essas limitações são contrabalanceadas pelos pontos fortes de nosso estudo: nós usamos um método automatizado para avaliar VGE, o que pode facilitar seu uso em grande escala, e nós tivemos um perfil cardiovascular abrangente dos indivíduos, avaliado por métodos padrões. Ainda, nós seremos capazes de acompanhar longitudinalmente esses indivíduos, o que trará novas perspectivas da relação do VGE com DAC. Finalmente, nós valíamos a relação entre VGE, CC e função endotelial, na tentativa de melhor compreender a associação do VGE com diferentes mecanismos envolvidos na DAC.

## Conclusão

No presente estudo, um VGE mais elevado foi associado com fatores de risco cardiovasculares e piores medidas da função endotelial. Além disso, o VGE não foi associado com CC em modelos multivariados. Nossos resultados geram a hipótese de que VGE elevado pode estar associado com DAC por uma via diferente do CC, que pode estar associada com disfunção endotelial, doença microvascular, e predominância de placas não calcificadas.

## *Material suplementar

Para informação adicional, por favor, clique aqui


