# Supplementary material for: Volume de Gordura Epicárdica está Associada com Disfunção Endotelial, mas Não com Calcificação Coronariana: Do ELSA-Brasil
Source: Arq Bras Cardiol. 2022 Sep 30;119(6):912–20. [Article in Portuguese] doi: 10.36660/abc.20210750 (PMC9814820; doi:10.36660/abc.20210750)
Supplement: Supplementary file 1 [file 2021-0750-suplementary-material.pdf]

SUPLEMENTARY MATERIAL

FIGURE

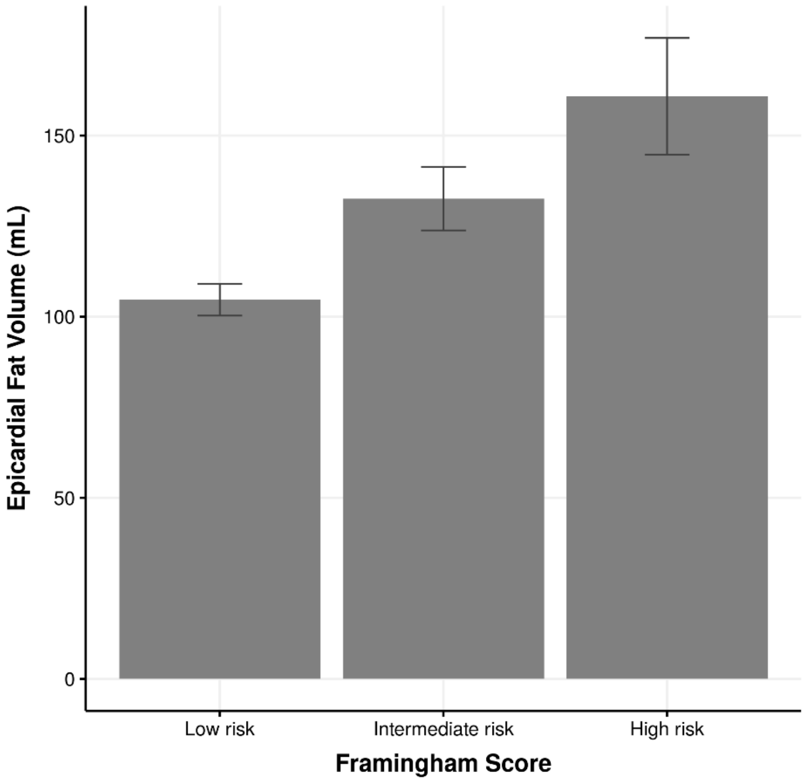

Supplementary Figure 1: Epicardial fat volume according to the *Framingham Risk Score* category for coronary artery disease in Brazilian adults. 0: low risk, 1; intermediate risk, 2: high risk. mL: milliliters. p <0.001 for comparison between all categories

TABLES

Supplementary Table 1 - Univariable association between cardiovascular risk factors and epicardial fat volume. Longitudinal Study of Adult Health (ELSA-Brasil), 2012-2016, (N = 470).

**Variable**	^{2}	**CI 95%**	**p-value**
**Sex** (reference men)	0.79	(0.74 – 0.84)	<0.001
**Age**	1.01	(1.01 – 1,02)	<0.001
**Race** (reference white)			
Black	0.84	(0.74 – 0.94)	0.002
Brown	0.90	(0.83 – 0.90)	0.015
**Schooling** (reference Elementary-school not completed)	1.26	(0.92 – 1.73)	0.146
Elementary-school completed	1.07	(0.82 – 1.40)	0.597
High-school	1.15	(0.89 – 1.48)	0.290
College			
**Physical activity status** (reference sedentary)			
Moderately active	1.01	(0.93 – 1.09)	0.890
Active	1.02	(0.85 – 1.23)	0.838
**BMI**	1.04	(1.03 – 1.05)	<0.001
**Waist circunference**	1.02	(1.016 – 1.021)	<0.001
**Smoking**	1.00	(0.87 – 1.15)	0.995
**Excessive drinker**	1.19	(1.06 – 1.33)	0.003

**Hypertension**	1.18	(1.10 – 1.27)	<0.001
**SBP**	1,00	(1,00 – 1,01)	<0,001
**Hypertension treatment**	1,18	(1,10 – 1,28)	<0,001
**Diabetes mellitus**	1,17	(1,06 – 1,28)	0.002
**Total/HDL cholesterol**	1.09	(1.05 – 1.13)	<0.001
**Tryglicerides**	1.001	(1.001 – 1.002)	<0.001
Framingham Score			
(Reference low risk)			
Intermediary risk	1.26	(1.16 – 1.36)	<0.001
High risk	1.49	(1.35 – 1.64)	<0.001

[2] exponencial regression coeficient. CI confidence interval, BMI body mass index, SBP systolic blood pressure

Supplementary Table 2 – Univariable association between cardiovascular risk factors and coronary calcium score. Longitudinal Study of Adult Health (ELSA-Brasil), 2012-2016 (N = 470).

**Variable**	**OR**	**IC 95%**	**p-value**
**Age**	1.08	(1.06 – 1.11)	<0.001
**Sex** (reference men)	0.37	(0.25 – 0.53)	<0.001
**Race** (reference whithe)	0.41		
Black	0.96	(0.21 – 0.76)	0.006
Brown		(0.64 – 1.44)	0.837
**Schooling** (reference	4.28		
Elementary-school not	1.94		
completed)	1.80	(0.85 – 26.27)	0.090
Elementary-school completed		(0.50 – 9.45)	0.361
High-school			0.400
College			
**Physical activity status**	1.12		
(reference sedentary)	2.02		
Moderately active		(0.76 – 1.64)	0.568
Active		(0.81 – 5.31)	0.136
**Smoking**	1.28	(0.63 – 2.58)	0.492
**Excessive drinker**	1.07	(0.58 – 1.95)	0.827
**BMI**	1.07	(1.03 – 1.12)	0.001
**Waist circuferance**	1.04	(1.03 – 1.06)	<0.001
**Diabetes Mellitus**	2.95	(1.82 – 4.95)	<0.001
**Hypertension**	2.58	(1.77 – 3.79)	<0.001

**SBP**	1.03	(1.02 – 1.05)	<0.001
**Hypertension treatment**	2.39	(1.62 – 3.54)	<0.001
**Total/HDL cholesterol**	1.39	(1.14– 1.69)	0.001
**Tryglicerides**	1.00	(1.00 – 1.01)	0.005
**Framingham Score**	2.73		
(Reference Low Risk)	7.60		
Intermediary risk		(1.76 – 4.26)	<0.001
High risk		(4.15 – 14.67)	<0.001
**EFV** (Reference q1)	1.34		
q2	1.88	(0.78 – 2.29)	0.294
q3	4.01	(1.11 – 3.23)	0.02
q4		(2.35 – 6.94)	<0.001

BMI body mass index, CI confidence interval, EFV epicardial fat volume OR Odds Ratio, SBP systolic blood pressure, EFV epicardial fat volume, q1 first quartil, q2 second quartil, q3 third quartil, q4 fourth quartil

Supplementary Table 3 – Univariable association between epicardial fat volume and endothelial function. Longitudinal Study of Adult Health (ELSA-Brasil), 2012-2016, (N=470).

**Variable**	**Pulse basal amplitude**		**PAT ratio**	
	^{2} **(CI)**	**p**	^{2} **(CI)**	**p**
**EFV (reference q1)**				
**q2**	1.39 (1.21 – 2.60)	<0.001	0.83 (0.77 -0.90)	<0.001
**q3**	1.83 (1.60– 2.12)	<0.001	0.79 (0.73 – 0.86)	<0.001
**q4**	2.02 (1.76 – 2.33)	<0.001	0.71(0.66 – 0.77)	<0.001

^{2} exponencial regression coeficient , CI confidence interval, *q1* first quartil, *q2* second quartil, *q3* third quartil, *q4* fourth quartil
