# Supplementary material for: Volume de Gordura Epicárdica está Associada com Disfunção Endotelial, mas Não com Calcificação Coronariana: Do ELSA-Brasil
Source: Arq Bras Cardiol. 2022 Sep 30;119(6):912–20. [Article in Portuguese] doi: 10.36660/abc.20210750 (PMC9814820; doi:10.36660/abc.20210750)
Supplement: Translation

# Translation: Epicardial Fat Volume Is Associated with Endothelial Dysfunction, but not with Coronary Calcification: From the Brazilian Longitudinal Study of Adult Health (ELSA-Brasil)

**Authors:** Karina P. M. P. Martins, Sandhi M. Barreto, Daniel Bos, Jesiana Pedrosa, Douglas R. M. Azevedo, Larissa Fortunato Araújo, Murilo Foppa, Bruce B. Duncan, Antonio Luiz P. Ribeiro, Luisa C. C. Brant

**Affiliations:** 1 Brazil Hospital das Clínicas, Universidade Federal de Minas Gerais, Belo Horizonte, MG – Brazil; 2 Brazil Faculdade de Medicina, Programa de Pós-Graduação, Belo Horizonte, MG – Brazil; 3 Brazil Departamento de Medicina Social e Preventiva da Universidade Federal de Minas Gerais, Belo Horizonte, MG – Brazil; 4 Netherlands Departamento de Epidemiologia, Erasmus MC – Netherlands; 5 Netherlands Departamento de Radiologia e Medicina Nuclear, Erasmus MC – Netherlands; 6 USA Departamento de Epidemiologia Clínica - Harvard TH Chan School of Public Health, Boston – USA; 7 Brazil Departamento de Anatomia e Imagem da Universidade Federal de Minas Gerais, Belo Horizonte, MG – Brazil; 8 Brazil Departamento de Estatística, Interno, Universidade Federal de Minas Gerais, Belo Horizonte, MG – Brazil; 9 Brazil Secretaria de Saúde Comunitária, Universidade Federal do Ceará, Fortaleza, CE – Brazil; 10 Brazil Hospital das Clínicas de Porto Alegre, Universidade Federal do Rio Grande do Sul, Porto Alegre, RS – Brazil; 11 Brazil Programa de Pós-Graduação, Universidade Federal do Rio Grande do Sul, Porto Alegre, RS – Brazil; 12 Brazil Departamento de Medicina Interna, Universidade Federal de Minas Gerais, Belo Horizonte, MG – Brazil

**Keywords:** Atherosclerosis, Intra-Abdominal Fat, Obesidade Abdominal

## Abstract

**Background:**

The increase in epicardial fat volume (EFV) is related to coronary artery disease (CAD), independent of visceral or subcutaneous fat. The mechanism underlying this association is unclear. Coronary artery calcium (CAC) score and endothelial dysfunction are related to coronary events, but whether EFV is related to these markers needs further clarification.

**Objectives:**

To evaluate the association between automatically measured EFV, cardiovascular risk factors, CAC, and endothelial function.

**Methods:**

In 470 participants from the Brazilian Longitudinal Study of Adult Health (ELSA-Brasil) with measures of EFV, CAC score and endothelial function, we performed multivariable models to evaluate the relation between cardiovascular risk factors and EFV (response variable), and between EFV (explanatory variable) and endothelial function variables or CAC score. Two-sided p <0.05 was considered statistically significant.

**Results:**

Mean age was 55 ± 8 years, 52.3% of patients were men. Mean EFV was 111mL (IQ 86-144), and the prevalence of CAC score=0 was 55%. In the multivariable analyses, increased EFV was related to female sex, older age, waist circumference, and triglycerides (p<0.001 for all). Higher EFV was associated with worse endothelial function: as compared with the first quartile, the odds ratio for basal pulse amplitude were (q2=1.22, 95%CI 1.07-1.40; q3=1.50, 95%CI 1.30-1.74; q4=1.50, 95%CI 1.28-1.79) and for peripheral arterial tonometry ratio were (q2=0.87, 95%CI 0.81-0.95; q3=0.86, 95%CI 0.79-0.94; q4=0.80, 95%CI 0.73-0.89), but not with CAC score>0.

**Conclusion:**

Higher EFV was associated with impaired endothelial function, but not with CAC. The results suggest that EFV is related to the development of CAD through a pathway different from the CAC pathway, possibly through aggravation of endothelial dysfunction and microvascular disease.

## Introduction

Visceral fat is the most studied ectopic fat deposit, and increased visceral adiposity is related to glucose intolerance, insulin resistance and cardiovascular diseases, independent of body mass index (BMI).^1^Epicardial fat shares many of the pathophysiological properties of other visceral fat deposits, but with additional potential effects on the coronary inflammatory and atherosclerotic process.^2^Researchers from “The Framingham Heart Study^3 , 4^and the “Multi-Ethnic Study of Atherosclerosis (MESA)^5 , 6^studied the association of epicardial fat volume (EFV) with cardiovascular risk factors and identified that EFV not only correlates with obesity and metabolic disorders, but also with the presence of hypertension and coronary artery disease (CAD). In a systematic review published in 2015, the authors described nine studies that evaluated the capacity of EFV to predict major cardiovascular events. Although the findings are not consistent for all studies, the majority suggest that EFV quantification is significantly associated with clinical outcomes.^7^

Recent studies have shown that epicardial fat deposits are associated with CAD but not with coronary artery calcium (CAC) score, which evaluates the calcification in coronary arteries and has been shown in large prospective studies to be associated with the risk of future cardiovascular events.^8^These studies suggested that EFV could be related to other mechanisms of plaque formation different from calcified plaques.^9 , 10^Nerlekar et al.^11^demonstrated, in a meta-analysis published in 2017, the progressive association between the presence of epicardial fat and high-risk atherosclerotic plaques, which are those with high lipid content, little calcification and a thin fibrotic cap.^11^Another study demonstrated that higher EFV was associated with vulnerability of plaques in the coronary arteries.^12^

Our objective was to evaluate the association between EFV with cardiovascular risk factors and subclinical markers of atherosclerosis – CAC and microvascular endothelial function, both predictors of cardiovascular events.^13 , 14^

## Methods

### Participants

Our sample included participants from the Brazilian Longitudinal Study of Adult Health (ELSA-Brasil), which aims to study the determinants of cardiovascular disease and diabetes in 15,105 Brazilian adults. The eligibility criteria included active or retired employees of five universities and one research institute, aged 35 to 74 years, who volunteered to participate. Further details of the study design were published elsewhere.^15^In the Investigation Center of ELSA-Brasil in Minas Gerais (3,115 participants), endothelial function assessment through peripheral arterial tonometry (PAT), and computed tomography (CT) for CAC evaluation were performed. The PAT exam was introduced partway through the baseline, resulting in 1,535 valid examinations.^16^Of these, 550 participants were randomly selected for reassessment of PAT on the same day of the CT, and 546 attended. EFV measurements were done in 501 randomly selected participants with valid CT scans and PAT using R Development Core Team software (2020) R. Thirty patients were excluded due to technical problems in the EFV analyses (n=4) and in PAT analyses (n=26) and one patient who had an EFV measurement considered outlier was excluded, leaving a final sample of 470 participants ( Figure 1 ).^16^ELSA-Brasil was approved by the Research Ethics Committees of the participating institutions and the National Research Ethics Committee (CONEP 976/2006). All participants signed an informed consent form.

**Figure 1:** Flowchart of Participants. CT: computed tomography; PAT: peripheral arterial tonometry; EFV: epicardial fat volume.

### Study protocol

Demographic variables were collected during the study baseline and clinical characteristics in the second visit. Age, sex, self-reported race, schooling, physical activity, obesity, central obesity, smoking, alcohol use, hypertension, diabetes mellitus (DM), dyslipidemia, hypertriglyceridemia, and the *Framingham risk score for CAD,* ^17^which estimates the probability of developing a coronary event in 10 years, were used in the analyses. Data collection followed the ELSA-Brasil protocol, whose details can be found elsewhere.^18 - 20^Physical activity was evaluated using the International Physical Activity Questionnaire-short form (IPAQ-SF),^21^in which each type of activity is weighed by its energy requirements defined in MET (metabolic equivalent of task). The physical activity time per week is then converted to MET minutes (MET-min/week). The participant is considered sedentary if the sum of MET-min/week is <600; moderately active if 600-3000 MET-min/week and active if > 3000 MET-min/week. Participants were classified as current smokers or non-smokers and, regarding alcohol use, they were classified as non-users, non-heavy drinkers or heavy (men with consumption ≥210g alcohol/week and women with consumption ≥140g alcohol/week) drinkers. Hypertension was determined by patient report, systolic blood pressure (SBP) ≥ 140 mmHg, diastolic blood pressure (DBP) ≥90 mmHg or use of antihypertensive medications. DM was determined by participant report, use of antidiabetic medication, or fasting glycemia ≥ 126 mg/dL, or glucose level ≥ 200 mg/dL after two hour of oral glucose overload, or glycated hemoglobin ≥ 6.5%. The Framingham risk score for CAD was used as a categorical variable, stratified into low (<10%), intermediate (10-20%) and high (>20%) cardiovascular risk.

### Assessment of EFV

We performed non-contrast enhanced, ECG-triggered cardiac CT scans to assess CAC and EFV, using a 64-slice CT scanner (Lightspeed, General Electric). Images were acquired during respiratory apnea for 8-12 seconds. EFV was quantified using a standardized, validated and fully automated method described by Shahzad et al.^22^In brief, this method included two phases: (1) heart segmentation, (2) quantification of EFV in mL. The segmentation of the heart was performed using multi-atlas segmentation and registration using the Elastix software described by Klein et al.^23^For the EFV quantification a window width range of -30 to -200 Hounsfield units was used. A manual calibration was carried out for the present study, using MeVisLab software for manual delimitation of the pericardial sac of 15 participants, as shown in ( Figure 2 ). The results were compared to those obtained by Elastix software and calibrated.

**Figure 2:** Manual calibration to assess epicardial fat volume in 15 participants of the ELSA-Brasil.

### CAC score measures

The images were transferred to a GE ADW 4.5 workstation and to the Imaging Server of the ELSA-Brasil, where CAC score was calculated by the Agatston method by a radiologist with 10 years of experience, blinded for clinical information.

### Endothelial function measures

PAT exam was performed by two certified examiners using the Endo-PAT2000 (Itamar Medical Ltd., Caesarea, Israel) on the same day of the CT.^16 , 24^Briefly, the cuff was placed in the participant’s nondominant arm, 2 cm above the cubital fossa, and PAT probes on each index finger. Baseline pulse amplitude (BPA) was measured for five minutes. Arterial flow was interrupted on one side for five minutes by inflating the cuff at suprasystolic pressure. After five minutes, the cuff was deflated to induce reactive hyperemia, and the PAT signal was recorded for another five minutes. The contralateral finger was used to control for systemic changes. Two variables from PAT were used: mean BPA, which reflects the basal vascular tone and is calculated by logarithmically transforming the mean BPA values of both arms, and the PAT ratio, which reflects the response to reactive hyperemia. PAT ratio is the ratio of pulse amplitude 90 to 120 seconds after cuff release to the mean BPA. This result is divided by the corresponding ratio from the control finger and transformed to its natural logarithm.

### Statistical analysis

Categorical variables were expressed as frequencies and percentages, continuous variables as mean ± standard deviation or median and interquartile range, according to the result of the Kolmogorov-Smirnov test. Due to the right-skewed distribution of EFV, the natural logarithm of EFV was used in the analyses in which EFV was the dependent variable (association with cardiovascular risk factors). In analyses in which EFV was the independent variable, quartiles of EFV were constructed (association with subclinical measures of atherosclerosis: CAC score and endothelial function). CAC score was dichotomized at 0 or >0, and endothelial function measurements were analyzed as continuous variables.

Statistical analyses were performed in three steps and through models adapted to the distribution of the response variables: 1- evaluation of the univariable and multivariable association between cardiovascular risk factors and EFV through linear regression; 2- evaluation of the univariable and multivariable association between EFV and CAC score through logistic regression; and 3- evaluation of the univariable and multivariable association between EFV and measures of endothelial function through linear regression.

The variables were considered in four multivariable models determined *a priori* and retained if they showed association in the univariable analyses with p <0.10, as follows: Model 1, adjusted for sex and age; Model 2: Model 1, plus race, and schooling; Model 3: Model 2 plus physical activity, BMI, waist circumference, and smoking; Model 4: Model 3 plus SBP, antihypertensive use, DM, total cholesterol/HDL, and triglycerides.

BMI and waist circumference were not included simultaneously in models 3 or 4, due to a collinearity with a variance inflation factor (VIF) close to 8. If both were statistically significant, waist circumference was included, because it is a measure of ectopic fat, like EFV. The *Framingham risk score for CAD* was analyzed separately because it already represents an assessment of CAD risk, incorporating the combined effect of several cardiovascular risk factors.

Two-sided p <0.05 was considered statistically significant. Due to the numbers of variables in the model, Bonferroni correction was performed and a p<0.0038 was considered statistically significant. All analyzes were performed using R Development Core Team software (2020).

## Results

Table 1 shows the characteristics of participants. Mean age was 55 ± 8 years, with 52.3% men. For race, 50.9% were white, 33.0% were mixed and 12.3% were black. A high proportion of sedentary (59.2%) and highly educated participants were observed, the latter reflecting the work of participants (university employees). Mean BMI was 26.9 ± 4.6 kg/m^2^and median waist circumference was 92 (84-101) cm. Median EFV was 111 (86-144) mL. A CAC=0 was detected in 261 (55.5%) participants. Mean BPA was 6.57 ± 0.62 and mean PAT ratio was 0.42 ± 0.34.

**Table 1:** Characteristics of study participants (n = 470)

Characteristcs	
**Age,** y	55 ± 8
**Sex,** men %	246 (52.3)
Race*, %	
Black	58 (12.3)
Brown	239 (50.9)
White	155 (33)
**Educational level,** %	
Incomplete elementary school	10 (2.1)
Complete elementary school	17 (3.6)
High school	87 (18.5)
University degree	356 (75.7)
**Physical activity status,** %	
Sedentary	278 (59.1)
Moderately active	172 (36.6)
Active	20 (4.6)
**Smoking,** %	34 (7.2)
**Excessive drinker,** %	48 (10.2)
**BMI,** kg/m^2^	26.9 ± 4.7
**Waist circunference,** cm	91.8 (84.4 – 100.7)
**Diabetes mellitus,** %	81 (17.2)
**Hypertension,** %	183 (38.9)
**SBP,** mmHg	121 ± 16
**Hypertension treatment,** %	159 (33.8)
Total/HDL cholesterol	3.84 ± 0.96
**Tryglicerides,** mg/dL	108 (79 – 155)
**CAC = 0** ^**†**^, %	261 (55.5)
**EFV,** mL	111 (86 -144)
**BPA**	657 ± 0.62
**PAT ratio**	0.42 + 0.34

* We excluded 13 participants (yellow and indigenous), as they represented a small sample; five participants did not provide the data, and †1 participant did not have data. BMI: body mass index; BPA: baseline pulse; CAC: coronary calcium score; HDL: high density lipoproteins; EFV: epicardial fat volume; BPA: baseline pulse amplitude; PAT: peripheral arterial tonometry; SBP: systolic blood pressure.

### Association between cardiovascular risk factors and EFV

The univariable association between cardiovascular risk factors and EFV is shown in Supplementary Table 1 . As the variable EFV was transformed to its natural logarithm, the increase of 0.1 in the coefficient of each explanatory variable indicates a 10.5% increase in EFV. Only smoking, physical activity and schooling were not statistically associated with EFV. Regarding race/skin color, black and mixed individuals showed significantly lower EFV than white individuals. An increase in EFV was observed with the progression of cardiovascular risk assessed by the Framingham Risk Score for CAD ( Supplementary Figure 1 ).

In the multivariable analysis ( Table 2 ), the following covariates remained associated with a higher EFV: male sex, olderfat volume age, waist circumference, and triglycerides. In the final model, black race remained associated with lower EFV.

**Table 2:** Linear regression models of the association between cardiovascular risk factors and epicardial fat volume

Variable	Model 1		Model 2		Model 3		Model 4	

	β	IC 95%	β	IC 95%	β	IC 95%	β	IC 95%
**Age**	1.01	(1.01 – 1.02) †	1.01	(1.01 – 1.02) †	1.01	(1.007 – 1.013) †	1.01	(1.01 – 1.02) †
**Sex** (reference men)	0.76	(0.71 – 0.82) †	0.77	(0.72 – 0.82) †	0.87	(0.82 – 0.93) †	0.87	(0.81 – 0.93) †
**Race** (reference white)								
Black	...	...	0,85	(0.77 – 0.95)*	0.83	(0.76 – 0.91) †	0.85	(0.77 – 0.93) †
Brown	...	...	0.92	(0.86 -0.99)*	0.93	(0.88 – 1.00)*	0.94	(0.88 -1.00)
**Waist circuference**	...	...	...	...	1.02	(1.01 – 1.02) †	1.02	(1.01 – 1.02) †
**Excessive drinker**					1.06	(0.97 – 1.17)	1.05	(0.95 – 1.15)
**Diabetes Mellitus**	...	...	...	...	...	...	0.96	(0.88 – 1.04)
**SBP**	...	...	...	...	...	...	1.00	(0.996 – 1.001)
**Hypertension treatment**	...	...	...	...	...	...	0.99	(0.92 – 1.06)
**Total/HDL Cholesterol**	...	...	...	...	...	...	0.98	(0.95 – 1.02)
**Tryglicerides**	...	...	...	...	...	...	1.00	(1.000 - 1.001)*

* p<0.05, †p< 0.001. β exponential regression coefficient. CI: confidence interval; SBP: systolic blood pressure; HDL: high density lipoproteins.

### Association between EFV and CAC

Regarding the association between EFV and CAC, the crude logistic analysis revealed increased chances of CAC>0 among people in the third and fourth quartiles of EFV. However, these associations lost statistical significance in the multivariable analysis ( Table 3 ) in all the models considered. The univariable analysis of CAC with cardiovascular risk factors is shown in Supplementary Table 2 .

**Table 3:** Logistic regression models of the association between quartiles of epicardial fat volume and presence of coronary calcium

Variable	OR	CI 95%	p-value
**Model 1 (reference first quartile)**			
Second quartile	1.07	(0.60 – 1.91)	0.823
Third quartile	1.19	(0.66 – 2.14)	0.565
Fourth quartile	1.72	(0.93 – 3.19)	0.082
**Model 2 (reference first quartile)**			
Second quartile	0.99	(0.54 – 1.80)	0.966
Third quartile	1.03	(0.56 – 1.90)	0.921
Fourth quartile	1.48	(0.79 -2.79)	0.221
**Model 3 (reference first quartile)**			
Second quartile	0.91	(0.50 – 1.68)	0.776
Third quartile	0.78	(0.41 – 1.49)	0.455
Fourth quartile	0.87	(0.41 – 1 .52)	0.709
**Model 4 (reference first quartile)**			
Second quartile	0.94	(0.50 – 1.74)	0.838
Third quartile	0.81	(0.42 – 1.59)	0.547
Fourth quartile	0.88	(0.41 – 1.87)	0.734

*OR Odds Ratio CI confidence interval. First quartile (22.8-86.2), Second quartile (86.2-112), Third quartile (112-144), Fourth quartile (144-331). Model 1: sex, age. Model 2: Model 1, plus race and schooling. Model 3: Model 2, plus physical activity level, BMI, waist circumference and smoking. Model 4: Model 3, plus SBP, antihypertensive use, DM, total cholesterol / HDL and triglycerides.*

### Association between EFV and endothelial function

In the univariable association ( Supplementary Table 3 ), we observed a statistically significant association of all quartiles of EFV with endothelial function measures. We also noticed a dose response gradient for EFV quartiles and measures of endothelial function: the mean BPA was progressively higher and PAT ratio lower – reflecting more impaired endothelial function – in the higher EFV quartiles ( Figure 3 ). In the multivariable analysis, the association remained statistically significant in all models ( Table 4 ).

**Figure 3:** Endothelial function measures (mean) according to the epicardial fat volume stratified in quartiles. PAT: peripheral arterial tonometry.

**Table 4:** Logistic regression models of the association between epicardial fat volume and endothelial function

Variable	Basal Pulse Amplitude		PAT ratio	p-value

	OR (CI 95%)	p-value	OR (CI95%)	
**Model 1** (reference first quartile)				
Second quartile	1.31 (1.14 – 1.49)	0.001	0.86 (0.79 – 0.93)	<0.001
Third quartile	1.63 (1.42 – 1.87)	< 0.001	0.83 (0.76 – 0.89)	<0.001
Fourth quartile	1.62 (1.40 – 1.88)	< 0.001	0.77 (0.71 – 0.84)	<0.001
**Model 2** (reference first quartile)				
Second quartile	1.22 (1.07 – 1.40)	0.003	0.87 (0.81 – 0.94)	<0.001
Third quartile	1.54 (1.35 – 1.77)	< 0.001	0.84 (0.77 – 0.91)	<0.001
Fourth quartile	1.54 (1.33 – 1.78)	< 0.001	0.79 (0.73 – 0.86)	<0.001
**Model 3** (reference first quartile)				
Second quartile	1.22 (1.06 – 1.39)	0.004	0.88 (0.81 – 0.95)	0.001
Third quartile	1.52 (1.32 – 1.75)	< 0.001	0.85 (0.78 – 0.92)	<0.001
Fourth quartile	1.49 (1.26 – 1.77)	< 0.001	0.80 (0.73 – 0.89)	<0.001
**Model 4** (reference first quartile)				
Second quartile	1.22 (1.07 – 1.40)	0.004	0.87 (0.81 – 0.95)	0.001
Third quartile	1.50 (1.30 – 1.74)	< 0.001	0.86 (0.79 – 0.94)	<0.001
Fourth quartile	1.50 (1.28 – 1.79)	< 0.001	0.80 (0.73 – 0.89)	<0.001

*OR Odds Ratio CI confidence interval. First quartile (22.8-86.2), Second quartile (86.2-112), Third quartile (112-144), Fourth quartile (144-331). Model 1: sex, age. Model 2: Model 1, plus race and schooling. Model 3: Model 2, plus degree of physical activity, BMI, waist circumference and smoking. Model 4: Model 3, plus SBP, antihypertensive use, DM, total cholesterol / HDL and triglyceride.*

## Discussion

The present study evaluated EFV by an automated method its association with cardiovascular risk factors and subclinical markers of atherosclerosis – CAC score and endothelial function in 470 participants of the ELSA-Brasil. The main findings of the present study were: 1) Association of EFV with most cardiovascular risk factors. In the multivariable analysis, higher EFV was seen for: male sex, older age, white race, and higher triglycerides and waist circumference; 2) EFV was not associated with the presence of CAC in multivariable models; 3) Increased EFV was associated with endothelial dysfunction in multivariable models, in a dose-response manner. Our findings generate the hypothesis that epicardial fat deposits may be linked to CAD through a distinct pathway from calcified plaques, but rather potentially related to endothelial dysfunction, microvascular disease and possibly predominantly lipidic non-calcified plaques.

First, median EFV was 111 (IQ 86-144) mL, which is comparable to the results observed by Bos et al. (101 mL, IQ 80-130),^25^and in the Framingham Heart Study (108 ± 40) mL,^10^suggesting that, although nutritional transition is in a slightly delayed stage in Brazil, comparing to European countries and the US, ectopic fat deposits appear to be present in a similar manner. Bos et al.,^25^using the same fully automated method described by Shahzad et al.,^22^evaluated the association between EFV, the presence of calcification in vessel beds and cardiovascular risk factors, in a cross-sectional analysis. The authors observed that an increase in EFV was associated with an increase in the volume of coronary and external carotid artery calcification, but only in men (difference in calcification volume with increase of one standard deviation of EFV: 0.12 [95% CI: 0.04, 0.19] and 0.14 [95% CI: 0.06, 0.22], respectively).^25^We did not find an association between EFV and CAC score after adjustment for cardiovascular risk factors. The distinct profile of the populations studied may explain the differences, since older and more women were evaluated by Bos et al. Regarding the results, we did not perform an analysis stratified by sex due to our smaller sample size.

In a more recent publication by Lee et al.^10^from the Framingham Heart Study, the association between EFV and CAC was assessed longitudinally^10^in 1,732 participants of the Offspring and Third Generation Cohorts (49.6% men, mean age 49.9 years), who were followed for 6.1 years. The study evaluated 1,024 participants with baseline CAC score =0 and 708 participants with baseline CAC score > 0. No association was observed between the increase in EFV and the progression of CAC score after adjustment for BMI, waist circumference and visceral adipose tissue, nor between incident CAC and EFV, what corroborates our findings of no association between higher EFV and CAC after adjusting for clinical variables.^10^The absence of association reported here was also described in a recent meta-analysis published by Mancio et al.,^9^which demonstrated that the association between EFV and CAC was not maintained in multivariable models, but higher EFV remained associated with obstructive or significant coronary stenosis, and major adverse cardiovascular events.^9^The authors hypothesize that EFV is associated with CAD through other mechanisms and forms of presentation that differ from the burden of calcified plaques. Another possible hypothesis is that the mechanism of association between EFV and CAD may express different moments in the natural history of the disease, being earlier as compared to CAC expression.^26^

In order to better understand the mechanism by which epicardial fat and CAD may be related, we further investigated the association of EFV with microvascular endothelial function.^27^We found that higher EFV was strongly associated with impaired microvascular function, even in multivariable models. The association between impaired endothelial function and higher EFV has been demonstrated in other studies. However, while all these studies have used flow-mediated dilation (FMD),^28 - 32^the method used to evaluate endothelial function in ELSA-MG was PAT. FMD differs from PAT in the vessels in which endothelial function is evaluated: while FMD evaluates it in the brachial artery - a conduit vessel - PAT assesses the microvasculature.^14 , 33 , 34^Considering that endothelial dysfunction is a predictor of cardiovascular events,^33 , 35^our results support the hypothesis that higher EFV is related to CAD by different pathways from the formation of calcified atherosclerotic plaques, including endothelial dysfunction, microvascular disease, and lipid-rich non calcified plaques. Due to the proximity of epicardial fat to the coronary arteries, the epicardial fat tissues may exert vasocrine effects on the vessels, when inflammatory mediators produced by epicardial fat act on the vessels, leading to endothelial dysfunction.^11^Given the possibility of our results represent an epiphenomenon, we performed models that tried to minimize this effect by adjusting for confounding variables.

Our study has some limitations. It is a cross-sectional study that precludes inferences about causality. However, this study is included in a cohort study, and the follow-up of participants for major adverse cardiovascular events will be possible in further publications. The sample size did not permit analysis of subgroups stratified by sex or obesity status as it represents a subsample of the large ELSA cohort. Furthermore, only microvascular endothelial function was studied and its evaluation in other arterial beds could complement our findings. However, microvascular endothelial function more strongly correlates with metabolic cardiovascular risk factors^14 , 34^– which, in turn, are more closely related to obesity phenotypes – as compared with endothelial function evaluated at conduit vessels.^34^In addition, we did not use the gold standard method to evaluate endothelial function because it is an invasive method. These limitations are counterbalanced by the strengths of our study: we used an automated method to evaluate EFV, which may facilitate its use on a large scale, and we had a comprehensive cardiovascular profile of the individuals, assessed by standardized methods. In addition, we will be able to longitudinally follow these individuals, which may bring further perspectives of the relation of EFV with CAD. Lastly, we evaluated the relationship between EFV, CAC and endothelial function, in attempt to better understand the association of EFV with the different mechanisms involved in CAD.

## Conclusion

In the present study, higher EFV was associated with cardiovascular risk factors and worse measures of endothelial function. In addition, EFV was not associated with CAC in multivariable models. Taken together, our results generate the hypothesis that higher EFV may be associated with CAD through a different pathway from CAC, potentially related to endothelial dysfunction, microvascular disease and predominance of non-calcified plaques.

## * Supplemental Materials

For additional information, please, click here
